# Protecting and rejuvenating ageing skin by regulating endogenous hyaluronan metabolism using adipose-derived stem cell-secreted siRNAs

**DOI:** 10.3389/fmed.2025.1529936

**Published:** 2025-04-29

**Authors:** Benben Sun, Yanqiu He, Lingzhu Zhang, Siyu Liu, Menghan Chen, Jinmeng Pan, Jingwen Fang, Ye Wang, Haiyue Jiang, Xia Liu, Chen-Yu Zhang, Jing Li

**Affiliations:** ^1^Nanjing Drum Tower Hospital Centre of Molecular Diagnostic and Therapy, State Key Laboratory of Pharmaceutical Biotechnology, Jiangsu Engineering Research Centre for MicroRNA Biology and Biotechnology, NJU Advanced Institute of Life Sciences (NAILS), School of Life Sciences, Nanjing University, Nanjing, China; ^2^Research Unit of Extracellular RNA, Chinese Academy of Medical Sciences, Nanjing, China; ^3^Plastic Surgery Hospital, Chinese Academy of Medical Sciences and Peking Union Medical College, Beijing, China

**Keywords:** skin aging, hyaluronan (HA), extracellular vesicles (EVs), adipose-derived stem cells (ADSCs), siRNA therapy

## Abstract

**Background:**

Loss of moisture is the primary cause of skin ageing and dysfunction. The skin’s hydration largely depends on hyaluronan (HA) and its ability to retain water. Ultraviolet (UV) irradiation, which accounts for 80% of skin ageing (commonly referred to as photoaging), gradually disrupts the balance of HA metabolism, leading to a reduction in HA levels, dehydration, and, ultimately, the formation of wrinkles.

**Methods:**

In this study, we develop an RNAi-based strategy to treat aged skin by modulating endogenous HA metabolism. Hyaluronidase 2 (HYAL2), an enzyme responsible for HA degradation, is selected as the therapeutic target, given its significant upregulation in photoaged skin. To deliver the siRNA targeting HYAL2 to the skin, human adipose-derived stem cells (ADSCs) are engineered to stably express and secrete HYAL2-targeting siRNAs (ADSC/siR^H^) via small extracellular vesicles (sEVs).

**Results:**

*In vitro* experiments demonstrate that ADSC-delivered siRNAs are successfully internalised by recipient cells, where they restore UV-induced HA reduction by inhibiting HYAL2 expression. *In vivo* experiments revealed that subcutaneous implantation of engineered ADSCs prior to UV exposure significantly protects mouse skin from accelerated HA degradation, helping to retain water content and prevent UV-induced dryness. Furthermore, the application of engineered ADSCs to aged mouse skin can markedly restore HA and water content, effectively smoothing deep wrinkles and improving skin appearance.

**Conclusion:**

We developed an effective biological strategy to combat skin ageing and damage by preserving endogenous HA levels, which could be applied for facial rejuvenation in the future.

## Introduction

Healthy, youthful skin not only plays a crucial cosmetic role in enhancing social interactions but also serves as a protective barrier, shielding the body from environmental irritants and pathogen invasion (1). As one ages, the skin gradually exhibits noticeable phenotypic, structural, and functional changes. Cutaneous ageing is a complex process driven by both intrinsic and extrinsic factors. Intrinsic skin ageing is a chronological and inevitable process, similar to the ageing of other internal organs (2). Additionally, skin ageing is accelerated by external factors such as sun exposure, poor nutrition, smoking, and air pollution (2). Notably, repeated and prolonged exposure to ultraviolet (UV) radiation is the primary cause of extrinsic skin ageing and premature skin ageing, also known as photoaging, thereby contributing to approximately 80% of facial ageing (3). Aged skin is characterised by collagen degradation, dryness, loss of elasticity, wrinkling, and skin atrophy (4).

Moisturization is recognised as a key factor in maintaining resilient skin. Skin hydration is highly associated with hyaluronan (HA), which has a remarkable capacity to bind and retain water molecules (5). HA, primarily produced by mesenchymal-origin cells such as fibroblasts, is a major component of the extracellular matrix (ECM). HA content is significantly higher in the dermis than in the epidermis (6). HA is a non-sulphated glycosaminoglycan (GAG) composed of repeating disaccharide units of D-glucuronic acid and N-acetyl-D-glucosamine, with a molecular weight ranging from 10^5^ to 10^7^ Da (7, 8). HA is synthesised by hyaluronan synthases (HAS) on the inner surface of the plasma membrane in fibroblasts and is then extruded through pore-like structures into the extracellular space (9). The turnover rate of HA is highly dynamic. Its degradation is mediated by hyaluronidase (HYAL), which hydrolyses the hexosaminidic β (1 → 4) linkages between the disaccharide units (10). Currently, six types of HYAL are identified in human. Particularly, HYAL2 hydrolyses high-molecular-weight HA into small fragments of approximately 20 kDa, serving as the major enzyme responsible for HA turnover in both the dermis and the epidermis (10).

UV-induced photoaging disrupts the balance of HA metabolism, leading to a gradual reduction in HA content and dehydration in human skin (11). While acute UV exposure (5 min to 1 h) increases HA deposition—a response similar to that in wound healing (5)—long-term photoaging results in decreased HAS expression and increased HYAL expression in the skin, ultimately impairing HA metabolism (11–13). Maintaining HA content has emerged as a promising therapeutic strategy for treating ageing skin. One of the most promising attempts is the supplementation of exogenous HA into the skin. However, despite its commercial use, this strategy faces challenges such as rapid HA clearance, abnormal accumulation, and potential inflammation (14). Therefore, targeting endogenous HA metabolism presents a novel opportunity for maintaining HA content and promoting skin hydration.

Adipose-derived stem cells (ADSCs) are a type of mesenchymal stem cells (MSCs) that show great promise for skin regeneration. Recently, extracellular vesicles (EVs) derived from ADSCs have been shown to inherit therapeutic properties from their parent cells, demonstrating similar effects on repairing skin defects, including wound healing (15), photoaging (16), pigmentation (17), and the regeneration of skin physiological function (18, 19). EVs are a heterogenous group of bilayer-membrane vesicles secreted by nearly all cell types, consisting mainly of large EVs (lEVs, also known as microvesicles) and small EVs (sEVs, also known as exosomes). It is well-established that EVs can transfer non-coding RNA cargo into recipient cells, where the EV-delivered non-coding RNAs function as endogenous RNAs, regulating gene expressions (20). Given this property, EVs are being explored as biological carriers with low toxicity and high immune compatibility for delivering therapeutic siRNAs for disease treatment (21).

In this study, to restore the balance of endogenous HA metabolism, we used RNA interference (RNAi) technology to suppress HYAL2 expression, which is significantly elevated during ageing (11). To deliver siRNA into the skin, we selected ADSCs as the cell chassis to express and produce siRNAs, utilizing their naturally released EVs to ensure the effective delivery of the therapeutic siRNAs into recipient cells. After implanting these ADSCs into the skin, the sEVs secreted from ADSCs continuously transfer HYAL2 siRNAs into HA-producing cells, rebalancing HA metabolism and significantly improving the morphology of aged skin. Together, our study presents a proof-of-concept strategy to combat skin damage and ageing by regulating endogenous HA content.

## Materials and methods

### Cell isolation and culture

Fat pad tissues from patients who underwent liposuction at Peking Union Medical College Hospital were used to separate human ADSCs, as previously described (22). These studies were approved by the Ethics Committee of the Chinese Academy of Medical Sciences and Peking Union Medical College Plastic Surgery Hospital and Institute (Approval No. 2017-37). Informed consent was obtained from all subjects involved in the study. After isolation, ADSCs were cultured in mesenchymal stem cell medium (ScienCell, CA, United States) supplemented with 15% foetal bovine serum (FBS, Gibco, MA, United States), 1% penicillin-streptomycin (P/S) in a 37°C humidified atmosphere containing 5% CO_2_. ADSCs from the second passage were used to identify biological characteristics, while third-and fourth-passage cells were used for lentivirus injections and sEV collection. Characterisation was performed by analysing standard marker expression and inducing adipogenic and osteogenic differentiation combined with a standard surface marker analysis, as previously reported. The mouse embryonic fibroblast cell line NIH/3T3 and human embryonic kidney cell line HEK293T were purchased from the Shanghai Institute of Cell Biology, Chinese Academy of Sciences (Shanghai, China). NIH/3T3 and HEK293T cells were cultured in high-glucose (4.5 g/L) DMEM (Gibco) supplemented with 10% FBS (Gibco), penicillin, and streptomycin. Human ADSCs and all cell lines were maintained at 37°C in a humidified atmosphere containing 5% CO_2_. All cell lines were identified by short tandem repeat (STR) profiling at the source. A regular mycoplasma test showed that both cell lines were mycoplasma negative.

### Differentiation of ADSCs

The adipocyte differentiation was performed using an adipogenic differentiation kit for human adipose-derived mesenchymal stem cells according to the manufacturer’s instructions (OriCell, Guangzhou, China). Osteoblast differentiation was performed using an osteogenic differentiation kit for ADSCs (OriCell, Guangzhou, China). Chondroblast differentiation was performed using a chondrogenic differentiation kit for ADSCs (Cyagen Biosciences, CA, United States). Briefly, cells were initially seeded on a 0.1% gelatine-coated 6-well plate (2 × 10^4^ cells/cm^2^). Then, when cells reached 100% confluence, they were treated with a differentiation medium for 3 weeks, followed by treatment with a maintenance medium for 3 weeks. Differentiation into adipocytes, osteoblasts, and chondroblasts was evaluated using Oil Red O staining (for lipid droplet visualization), Alizarin Red staining (for calcium deposition visualization), and Alcian Blue staining (for proteoglycan detection), respectively.

### sEV isolation and identification

sEVs derived from ADSCs were extracted from the collected supernatant, as previously described. Briefly, the supernatant was collected from ADSCs and centrifuged at 800 × g for 10 min to remove dead cells. Then, the supernatant was collected and centrifuged at 3,000 × g for 20 min to remove cell debris. The supernatant containing the cell-free medium was transferred to a new tube and centrifuged at 10,000 × g to remove large EVs. Then, an exosome isolation kit (Vazyme, Nanjing, China) was used to isolate sEVs according to the manufacturer’s instructions. The sEV pellet was resuspended in PBS for cell incubation assay or in RIPA lysis buffer for the western blotting analysis. A nanoparticle tracking analyser (Nanosight LM10, Wiltshire, United Kingdom) was used to determine the size distribution and particle number of isolated vesicles. Marker expressions were analysed using the western blotting analysis.

### Plasmid construction, lentivirus construction, and engineered ADSCs

siRNA sequence was synthesized by GenScript (Nanjing, China) and cloned into the pLKO.1 vector (GenScript). To construct the lentivirus expressing HYAL2 siRNAs, the plasmid expressing HYAL2 siRNAs was transfected into HEK293T cells together with plasmids psPAX2 and pMD2G using Lipofectamine^™^ 2000 Transfection Reagent (Invitrogen, CA, United States) at a molar ratio of 10:5:1. Transfections were performed according to the manufacturer’s instructions. Lentivirus particles were collected from the medium supernatant after 48 h and filtered using a 0.22-μm filter. For viral infection, ADSC cells at the third or fourth passage were incubated with HYAL2 siRNA-expressing lentivirus in the presence of 4 μg/mL of polybrene (Sigma-Aldrich, MO, United States). Afterwards, cells were cultured as a polyclonal population and maintained under puromycin (Gibco). Polyclonal cells were used for sEV isolation. siRNA sequences are listed in the Supplementary material.

### Animals

Six-week-old female BALB/c nude mice were purchased from GemPharmatech (Nanjing, China). The animals were housed under specific-pathogen-free (SPF) conditions and maintained in a temperature-controlled room with a 12-h light/dark cycle. The animal studies were reviewed and approved by the Animal Ethical and Welfare Committee of Nanjing University (Nanjing, China, Approval Number: IACUC-2206003-1). Animal studies were designed and conducted following the Animal Research: Reporting of *In Vivo* Experiments (ARRIVE) guidelines.

### The model of UV irradiation-induced skin damage and ageing

Minimal erythema dose (MED) was first defined (60 mJ/cm^2^), and the irradiation energy was adjusted according to the MED value. To evaluate the protective effect of ADSC-delivered siRNAs, 1 × 10^6^ ADSCs were subcutaneously implanted into the dorsal skin prior to UV irradiation. Then, mice were subjected to acute UVB irradiation with an intensity of five MED (300 mJ/cm^2^) in total within 5 days, as previously reported (23). Samples were collected the day after the final irradiation. To evaluate the rejuvenation effect of ADSC-delivered siRNAs, UVB-induced skin ageing was conducted as previously described. Briefly, mice were subjected to UVB irradiation for 10 weeks with an intensity of 130 MED in total (24). During the first 4 weeks, mice were subjected to UVB irradiation with an intensity of two MEDs (120 mJ/cm^2^) each week, followed by three MED (180 mJ/cm^2^) in weeks 5 to 10. After irradiation, 1 × 10^6^ ADSCs were subcutaneously injected around the irradiated dorsal skin. After 1 week, mice were euthanized for sampling and analysis. Mice were irradiated under a UVB lamp (Philips, 311 nm, Germany, TL20W/01). The UVB irradiated dose was calculated according to the energy density measured with a UVB energy detector (Tenmars, Nanjing, China).

### Gross assessment of the skin

Photos of the dorsal skin were taken, and wrinkle scores were evaluated using a classical clinical wrinkle score system (25). Skin wrinkles, water content, and elasticity of the skin were recorded by a multifunctional skin detector (Dermoscopy skin analyser, Wuhan, China).

### Histological analysis

Skin specimens taken from the UV-exposed area of the mouse dorsum were fixed in 4% paraformaldehyde (PFA) and embedded in paraffin. Sections obtained using the microtome were collected on glass slides and stained with haematoxylin and eosin (H&E) and Masson’s trichrome staining by Hematoxylin-Eosin (HE) Stain Kit (Solarbio, Beijing, China) and Masson’s Trichrome Stain Kit (Solarbio, Beijing, China). Images were taken using light microscopy (Nikon, Japan), and the thicknesses of the epidermis and the dermis were measured using Image-Pro Plus software (Rockville, MD, United States), as previously described (26).

### Immunofluorescence staining of HA

Cryo-sections were fixed in 4% PFA for 10 min and rinsed with PBS, followed by blocking with 5% BSA (Sigma-Aldrich) for 1 h. Sections were then incubated with HA antibody (GeneTex, TX, United States) at 4°C overnight. Then, sections were rinsed with PBS and incubated with a fluorescently labelled secondary antibody (Alexa Fluor^™^ Plus 594, Invitrogen) for 1 h. Then, sections were rinsed and placed in a DAPI staining solution for 10 min. After being rinsed in PBS, the sections were ready for examination. Slides were imaged using the confocal imaging system (TCS SP8-Maitai M, Leica, Wetzler, Germany), and images were processed using TCS SP8 software (Leica). The results are from three independent experiments.

### RNA extraction and qRT-PCR

TRIzol reagent (TaKaRa, Dalian, China) was used to extract total RNA from cells, skin tissues, and sEVs according to the manufacturer’s instructions. Customized TaqMan siRNA probes were used for siRNA quantification according to the manufacturer’s instructions. cDNA was synthesised from 1 μg of total RNA using AMV reverse transcriptase and RT-primer (Applied Biosystems, CA, United States). qRT-PCR was performed using TagMan^™^ MicroRNA assay (Applied Biosystems) on the ABI 7300 system (Applied Biosystems). To determine the absolute levels of siRNAs, a series of synthetic single-stranded siRNAs with known concentrations were amplified to generate a standard curve. siRNA concentrations were calculated by referring to the standard curve and presented as the absolute amount of siRNA in 1 g total RNA (pmol/g total RNA). For mRNA quantification, 1 μg of total RNA was reverse transcribed into cDNA using Oligo(dT). Then, specific forward and reverse primers were used for mRNA amplification on the ABI 7300 system (Applied Biosystems). mRNA expression levels were normalized to 18S RNA. All reactions were run in triplicate. Ct values were determined using fixed threshold settings. Primer sequences are listed in the Supplementary material.

### Western blotting analysis

Total protein from cells, tissues, or sEVs was extracted using RIPA lysis buffer (Beyotime, Shanghai, China). Protein concentrations were determined with a BCA assay kit (Vazyme). Lysates were used for electrophoresis on SDS-PAGE (10%) gel and transferred to polyvinylidene fluoride (PVDF) membranes, which were then blocked with skim milk for 1 h and incubated with primary antibodies, followed by incubation with a secondary antibody at room temperature for 1 h. The antibodies used in the experiments were as follows: HYAL2 antibody (Bioss, MA, United States), α-tubulin antibody (Cell Signalling Technology, MA, United States), β-actin antibody (Cell Signalling Technology), CD63 antibody (Santa Cruz, TX, United States), ALIX antibody (Proteintech, CA, United States), and CD9 antibody (Abcam, Cambridge, United States). Blots were detected using an enhanced chemiluminescence kit (Vazyme) and analysed by a western blotting imaging system (Tanon 5200, Shanghai, China).

### Statistical analysis

Data are means with error bars showing SEM. Technical and biological triplicates of each experiment were performed. All statistical analyses were performed using commercially available software (GraphPad Prism 9). Details of the statistics for each figure can be found in the figure legends. A *p*-value of <0.05 was considered statistically significant.

## Results

### Generation of engineered ADSC-expressing therapeutic siRNAs targeting HYAL2

**Figure 1:**
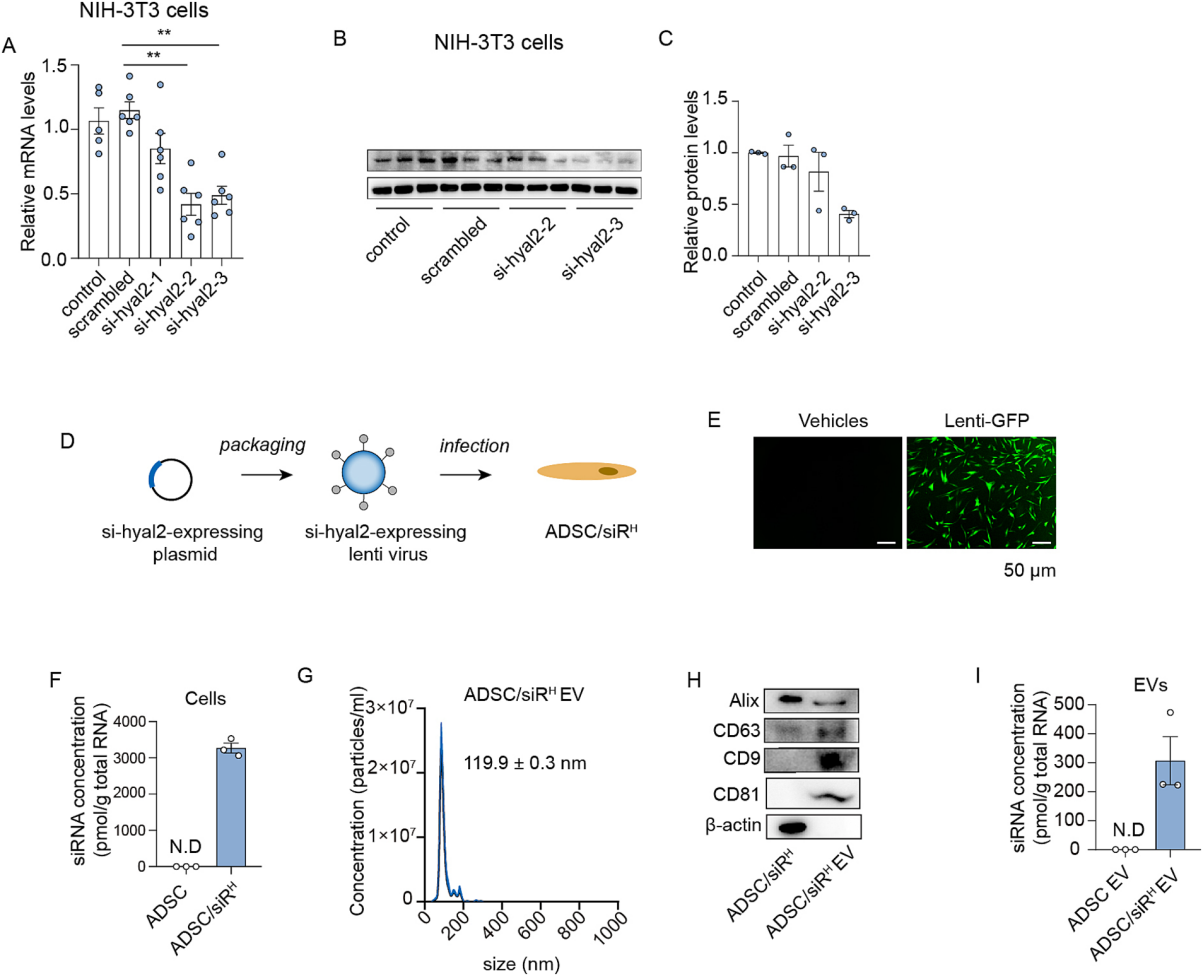
**(A)** qPCR analysis of HYAL2 mRNA levels in untreated NIH/3T3 cells or NIH/3T3 cells transfected with scrambled RNA, si-hyal2-1, si-hyal2-2, and si-hyal2-3 (*n* = 6). **(B,C)** A western blotting analysis of HYAL2 protein levels in untreated NIH/3T3 cells or NIH/3T3 cells transfected with scrambled RNA, si-hyal2-2, and si-hyal2-3 (*n* = 3). **(D)** Schematic of the generation of engineered ADSCs. **(E)** The infection efficiency was expressed by lenti-GFP in NIH/3T3 cells. **(F)** qPCR analysis of si-hyal2 concentration in ADSCs or ADSC/siR^H^ (*n* = 3). **(G)** Nanoparticle tracking analysis (NTA) of sEVs isolated from the medium of ADSC/siR^H^. **(H)** Representative western blot analysis of the sEV membrane markers CD63, CD9, CD81, and proteins that mediate the budding and abscission processes during sEV exocytosis in ADSC/siR^H^ and their sEVs. **(I)** qPCR analysis of si-hyal2 concentrations in sEVs isolated from the medium of ADSCs and ADSC/siR^H^ (*n* = 3). The results represent the mean ± SD; *p*-values are calculated using one-way ANOVA; ^**^p < 0.001.

To identify siRNAs that efficiently inhibit HYAL2 expression, three siRNA sequences were designed and synthesised. The siRNAs were transfected into NIH/3T3 cells, which possess the protein machinery for HA turnover. The results showed that siRNA 3 (si-hyal2-3) exhibited the highest interference efficiency, as assessed by HYAL2 mRNA and protein levels (Figures 1A–C). Therefore, si-hyal2-3 was selected for the generation of engineered ADSCs. Next, ADSCs were isolated from human subcutaneous fat pads. Flow cytometry analysis revealed that the majority of isolated cells expressed essential ADSC markers CD73 (92%), CD105 (98%), and CD90 (98%), while over 99% of cells were negative for CD34, CD11b, CD19, CD45, and HLA-DR (Supplementary Figures S1A–D) (27). Additionally, the cells exhibited the capabilities to differentiate into adipocytes, osteoblasts, and chondroblasts, further confirming their identity as ADSCs (Supplementary Figures 1E,F) (27). These results indicate that the isolated cells can be classified as ADSCs. Next, the si-hyal2-3-expressing plasmids were packaged into lentiviruses, which were then transfected into ADSCs to generate engineered ADSCs that stably express HYAL2 siRNAs (ADSC/siR^H^) (Figure 1D). GFP protein was used as a readout for the infection, and the results showed efficient lentivirus infection in ADSCs (Figure 1E). Consistently, a substantial level of siRNAs was detected in ADSC/siR^H^ (Figure 1F). Next, sEVs derived from ADSCs were isolated from the culture medium, as previously described. The sEVs were characterized in terms of particle diameter and surface markers. The mean particle diameters were approximately 119.9 nm, and essential surface markers (CD63, CD9, and CD81), as well as proteins involved in sEV biogenesis, were detected (Figures 1G,H). These results confirm that we successfully isolated sEVs derived from ADSC/siR^H^. Furthermore, we detected abundant levels of siRNAs in the sEVs derived from ADSC/siR^H^ (Figure 1I). Collectively, these findings demonstrate that we obtained engineered ADSCs to release siRNAs targeting HYAL2.

### Small EV-derived ADSC/siR^H^ effectively suppress HYAL2 expression and preserve HA content in NIH/3T3 cells

**Figure 2:**
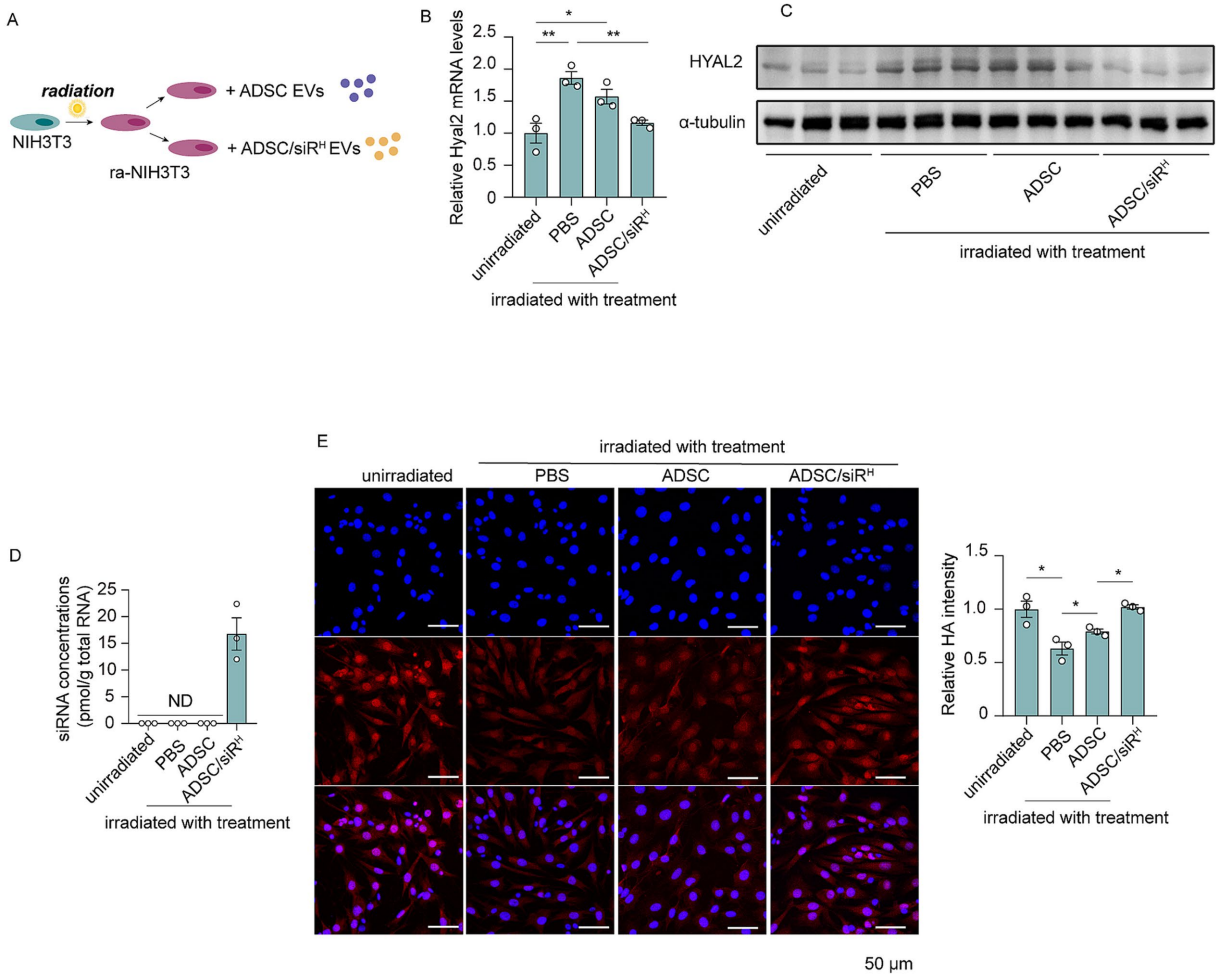
**(A)** Schematic of NIH/3T3 cells treated with irradiation and sEVs. **(B)** qPCR analysis of HYAL2 mRNA levels in unirradiated NIH/3T3 cells and irradiated NIH/3T3 cells treated with PBS, sEVs derived from ADSCs, and sEVs derived from ADSC/siR^H^ (*n* = 3). **(C)** A western blotting analysis of HYAL2 protein levels in unirradiated NIH/3T3 cells and irradiated NIH/3T3 cells treated with PBS, sEVs derived from ADSCs, and sEVs derived from ADSC/siR^H^ (*n* = 3). **(D)** qPCR analysis of si-hyal2 concentrations in unirradiated NIH/3T3 cells and irradiated NIH/3T3 cells treated with PBS, sEVs derived from ADSCs, and sEVs derived from ADSC/siR^H^ (*n* = 3). **(E)** The representative immunofluorescence images and fluorescence intensity analysis of HA (red) in unirradiated NIH/3T3 cells and irradiated NIH/3T3 cells treated with PBS, sEVs derived from ADSCs, and sEVs derived from ADSC/siR^H^, and blue staining (DAPI) represents nuclei. The results represent the mean ± SD; *p*-values are calculated using a one-way ANOVA; ^*^p < 0.05 and ^**^p < 0.01.

Next, we investigated whether HYAL2 siRNAs derived from ADSC/siR^H^ could regulate HA metabolism in recipient cells. NIH/3T3 cells were subjected to UVB irradiation to induce an imbalance in HA metabolism, followed by treatment with sEVs derived from ADSCs or ADSC/siR^H^ (Figure 2A). Compared to unirradiated NIH/3T3 cells, UVB irradiation significantly increased both HYAL2 mRNA and protein levels (Figures 2B,C). As anticipated, HYAL2 expression was suppressed by sEVs derived from ADSC/siR^H^, whereas PBS or sEVs derived from wild-type ADSCs did not affect HYAL2 levels (Figures 2B,C). Furthermore, a substantial level of HYAL2 siRNA was detected in NIH/3T3 cells treated with sEVs from ADSC/siR^H^ (Figure 2D). Consistent with these results, HA content was significantly reduced following UVB irradiation, suggesting that UVB-induced HYAL2 overexpression accelerates HA degradation and disrupts HA metabolism (Figure 2E). Treatment with sEVs derived from ADSC/siR^H^ restored HA content to levels comparable to those in unirradiated cells, while PBS and wild-type ADSC treatments did not affect HA content (Figure 2E). These findings demonstrate that sEV-delivered siRNAs effectively suppress UVB-induced HYAL2 upregulation and restore HA content.

### ADSC/siR^H^-derived siRNAs play a protective role in UV-induced skin damage by inhibiting HA degradation

Next, we aim to investigate whether treatment with ADSC/siR^H^ could have an anti-ageing effect *in vivo*. ADSC/siR^H^ were administered subcutaneously on the dorsal side, and we found that a substantial level of siRNAs remained detectable in the skin for up to 14 days. This finding indicates that ADSC/siR^H^ has a stable, long-term capacity for siRNA release (Supplementary Figure 2).

**Figure 3:**
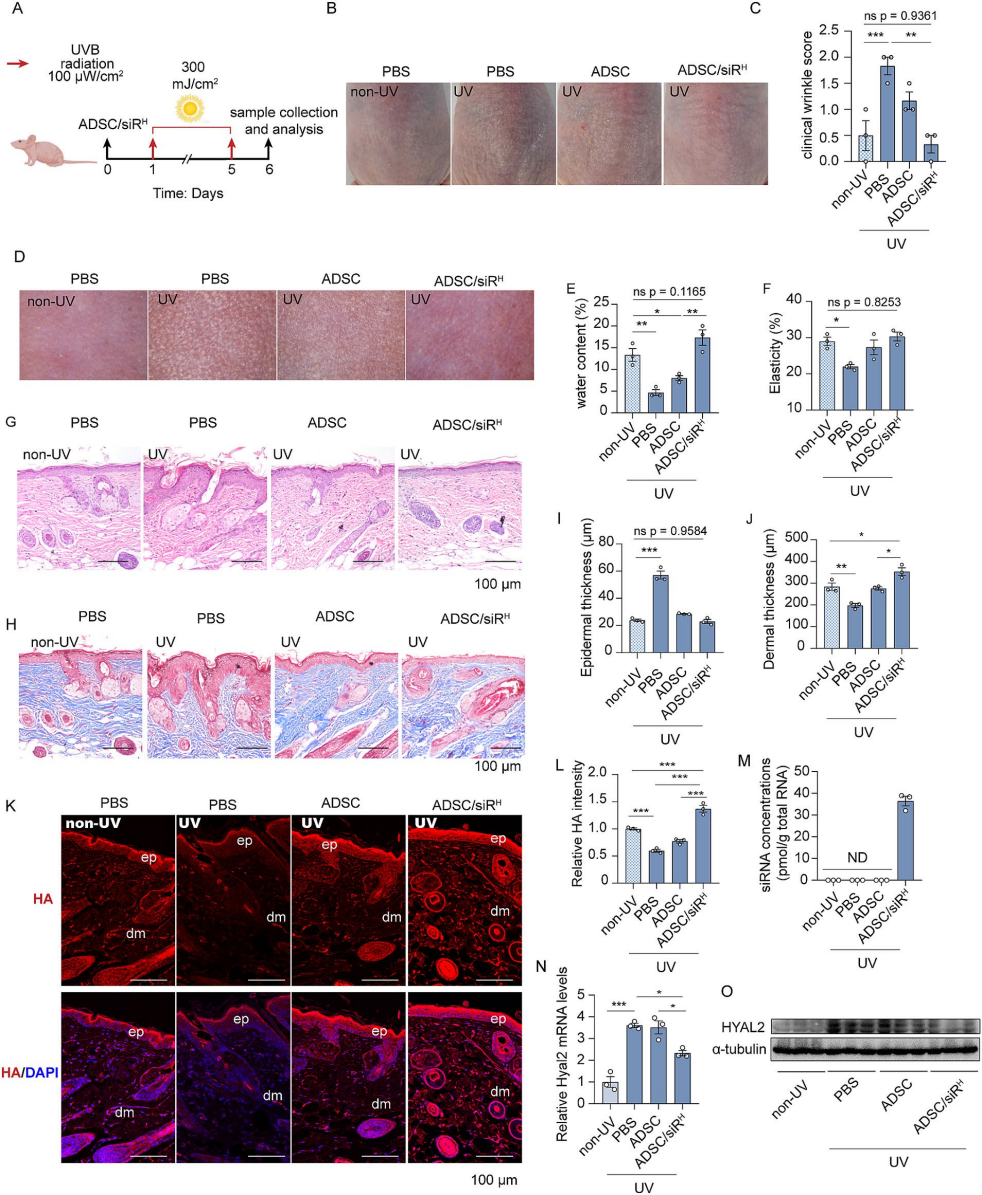
**(A)** A schematic design of the generation of the photodamage mouse model and ADSC/siR^H^ treatment. **(B)** The representative dorsal skin photos of the unirradiated mice and the irradiated mice treated with PBS, ADSCs, and ADSC/siR^H^. **(C)** The wrinkle analysis of the unirradiated mice and the irradiated mice treated with PBS, ADSCs, and ADSC/siR^H^ by clinical wrinkle score (*n* = 3). **(D–F)** The representative dorsal skin photos **(D)**, water content **(E)**, and elasticity analysis **(F)** by Skin Analyzer of the unirradiated mice and the irradiated mice treated with PBS, ADSCs, and ADSC/siR^H^ (*n* = 3). **(G,I)** The representative histochemistry staining images **(G)** and epidermal thickness analysis **(I)** of the dorsal skin tissue of the unirradiated mice and the irradiated mice treated with PBS, ADSCs, and ADSC/siR^H^ (*n* = 3). **(H,J)** The representative Masson trichrome staining images **(H)** and dermal thickness analysis **(J)** of the dorsal skin tissue of the unirradiated mice and the irradiated mice treated with PBS, ADSCs, and ADSC/siR^H^ (*n* = 3). **(K,L)** The representative immunofluorescence images **(K)** and fluorescence intensity analysis of HA (red) **(L)** of the dorsal skin tissue of the unirradiated mice and the irradiated mice treated with PBS, ADSCs, and ADSC/siR^H^, and blue staining (DAPI) represents nuclei (*n* = 3). **(M)** qPCR analysis of si-hyal2 concentrations in the dorsal skin tissue of the unirradiated mice and the irradiated mice treated with PBS, ADSCs, and ADSC/siR^H^ (*n* = 3). **(N)** qPCR analysis of HYAL2 mRNA levels in the dorsal skin tissue of the unirradiated mice and the irradiated mice treated with PBS, ADSCs, and ADSC/siR^H^ (*n* = 3). **(O)** A western blotting analysis of HYAL2 protein levels in the dorsal skin tissue of the unirradiated mice and the irradiated mice treated with PBS, ADSCs, and ADSC/siR^H^ (*n* = 3). The results represent the mean ± SD; *p*-values are calculated using a one-way ANOVA; ^*^p < 0.05, ^**^p < 0.01, and ^***^p < 0.001.

Protecting the skin from daily photo damage is critical for maintaining skin hydration and a youthful appearance. We investigated whether pre-administration of ADSC/siR^H^ could prevent UV-induced HA degradation. To test this, mice were first injected subcutaneously with ADSC/siR^H^ on the dorsal side, followed by short-term acute UVB irradiation (300 mJ/m^2^ for five consecutive days) (Figure 3A). Morphological analysis revealed that UVB irradiation induced fine wrinkle formations and skin dryness in comparison to unirradiated mice (Figures 3B,C). However, UVB-induced wrinkle formations were mitigated by ADSCs and, more importantly, completely prevented by ADSC/siR^H^ treatment compared to mice treated with PBS (Figures 3B,C). Consistently, the dermatoscopic analysis revealed that UVB irradiation resulted in significant roughness and wrinkles, while ADSC/siR^H^ treatment dramatically reduced wrinkles and smoothed the skin. In line with these findings, skin water content and elasticity were severely compromised by UVB irradiation but fully preserved with ADSC/siR^H^ treatment to levels comparable to those in healthy skin (Figures 3D–F). Histochemical staining and Masson’s trichrome staining consistently showed that ADSC/siR^H^ treatment reduced epidermal thickness and improved dermal thickness (Figures 3G–J). Next, we analysed HA contents and HYAL2 expression levels in mouse skin. HA content was significantly reduced by UVB irradiation (Figures 3K,L). Meanwhile, we observed that HYAL2 expression levels were significantly increased following UVB irradiation, indicating enhanced HA degradation in skin exposed to short-term acute UVB (Figures 3N,O). Interestingly, injection of ADSC/siR^H^ not only prevented UV-induced HA degradation but also slightly increased HA content in the skin (Figures 3K,L). Furthermore, substantial levels of HYAL2 siRNAs were detected in the skin of mice treated with ADSC/siR^H^, resulting in the suppression of HYAL2 expression (Figures 3M–O). Taken together, these results indicate that preventing HA degradation by targeting HYAL2 provides a protective role in skin exposed to acute photo-damage. Moreover, while pre-treatment with ADSCs showed protective effects on skin texture, as evidenced by skin imaging analysis (Figure 3B), wrinkle score analysis (Figure 3C), and elasticity analysis (Figure 3F), the treatment with ADSC/siR^H^ exhibited a more significant protective effect. This finding suggests that specifically targeting HA degradation has a more pronounced anti-ageing effect on the skin.

### ADSC/siR^H^-derived siRNAs rejuvenate aged skin by inhibiting HA degradation

**Figure 4:**
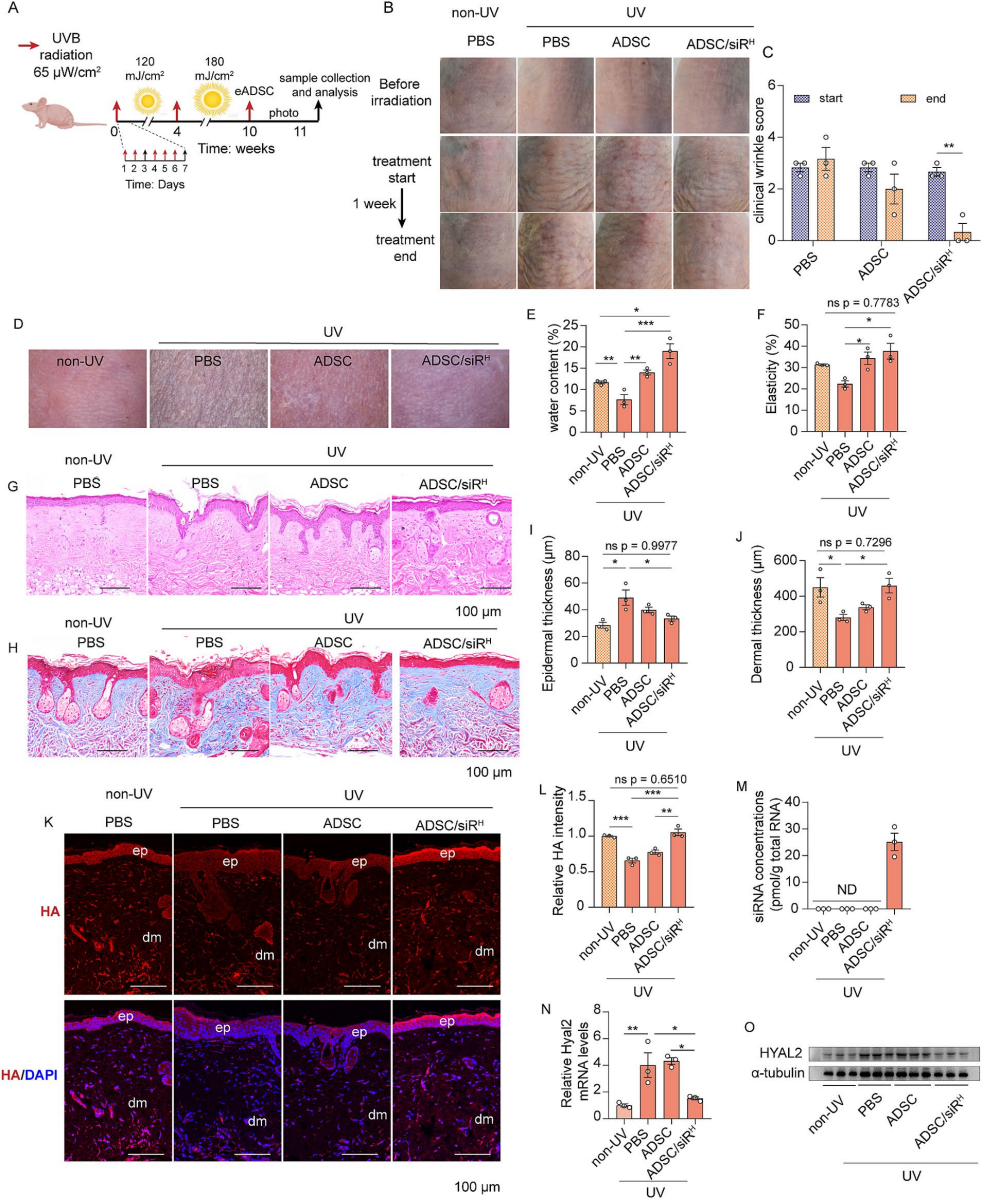
**(A)** Schematic of the generation of the skin ageing mouse model and ADSC/siR^H^ treatment. **(B)** The representative dorsal skin photos of the unirradiated mice and the irradiated mice treated with PBS, ADSCs, and ADSC/siR^H^. **(C)** The wrinkle analysis of the irradiated mice treated with PBS, ADSCs, and ADSC/siR^H^ before and after treatment by clinical wrinkle score (*n* = 3). **(D–F)** The representative dorsal skin photos **(D)**, water content **(E)**, and elasticity analysis **(F)** by Skin Analyzer of the unirradiated mice and the irradiated mice treated with PBS, ADSCs, and ADSC/siR^H^ (*n* = 3). **(G,I)** The representative histochemistry staining images **(G)** and epidermal thickness analysis **(I)** of the dorsal skin tissue of the unirradiated mice and the irradiated mice treated with PBS, ADSCs, and ADSC/siR^H^ (*n* = 3). **(H,J)** The representative Masson’s trichrome staining images **(H)** and dermal thickness analysis **(J)** of the dorsal skin tissue of the unirradiated mice and the irradiated mice treated with PBS, ADSCs, and ADSC/siR^H^ (*n* = 3). **(K,L)** The representative immunofluorescence images **(K)** and fluorescence intensity analysis of HA (red) **(L)** of the dorsal skin tissue of the unirradiated mice and the irradiated mice treated with PBS, ADSCs, and ADSC/siR^H^, and blue staining (DAPI) represents nuclei (*n* = 3). **(M)** qPCR analysis of si-hyal2 concentrations in the dorsal skin tissue of the unirradiated mice and the irradiated mice treated with PBS, ADSCs, and ADSC/siR^H^ (*n* = 3). **(N)** qPCR analysis of HYAL2 mRNA levels in the dorsal skin tissue of the unirradiated mice and the irradiated mice treated with PBS, ADSCs, and ADSC/siR^H^ (*n* = 3). **(O)** A western blotting analysis of HYAL2 protein levels in the dorsal skin tissue of the unirradiated mice and the irradiated mice treated with PBS, ADSCs, and ADSC/siR^H^ (*n* = 3). The results represent the mean ± SD, and *p*-values are calculated using a one-way ANOVA; ^*^p < 0.05, ^**^p < 0.01, and ^***^p < 0.001.

Long-term UVB exposure is a major extrinsic factor in skin ageing, accounting for 80% of facial ageing. We then sought to investigate whether ADSC/siR^H^ could rejuvenate aged skin by rebalancing HA metabolism. To mimic the natural process of skin ageing, mice were exposed to UVB irradiation for 10 weeks, with irradiation occurring 5 days per week (Figure 4A). Morphological images revealed classic features of aged skin on the dorsal skin of mice, including deep and pronounced wrinkles (Figure 4B). Consistently, wrinkle score analysis demonstrated that UVB irradiation induced significant wrinkle formation (Figure 4C). These results indicate that we successfully generated a mouse model of aged skin. Next, ADSCs or ADSC/siR^H^ were implanted dorsally in the area previously exposed to irradiation, with PBS injected as a control treatment. After 1 week, wrinkles of mice treated with ADSCs were reduced (Figures 4B,C). More importantly, treatment with ADSC/siR^H^ resulted in a significantly greater reduction in wrinkles on aged skin (Figures 4B,C). The dermatoscopic analysis further revealed that ADSC/siR^H^ treatment also restored skin water content and elasticity, dramatically improving skin texture (Figures 4D–F). Aged skin typically features increased epidermal thickness and reduced dermal thickness. Histological analysis, including H&E and Masson’s trichrome staining, showed that epidermal thickness was significantly increased, and dermal thickness was increased following ADSC/siR^H^ treatment, with both parameters restored to levels comparable to skin from non-UV-treated mice (Figures 4G–J). These results indicate that ADSC/siR^H^ treatment improves the architecture of aged skin. Next, we investigated HA content and HYAL2 levels. The result showed that the HA content was significantly restored to levels comparable to those in healthy skin following ADSC/siR^H^ treatment (Figures 4K,L). This restoration was associated with a significant reduction in HYAL2 expression (Figures 4N,O), which was suppressed by the siRNAs released from ADSC/siR^H^ (Figure 4M). Together, these results indicate that ADSC/siR^H^-derived siRNA can rejuvenate aged skin by rebalancing HA metabolism.

## Discussion

People across the world are seeking ways to maintain youthful facial skin. Youthful skin is primarily characterised by its high water content, and skin ageing is strongly associated with the loss of skin hydration (5). Therefore, compared to other widely used strategies such as retinoids and laser therapy, maintaining moisture in the skin may offer a more effective and crucial point-targeted strategy to prevent skin ageing. As a critical water-binding molecule in the skin, HA content has long been considered a promising target for skin rejuvenation. Supplementation of HA through daily cosmetic products offers a convenient way to maintain HA levels in the skin. However, despite claims of enhanced hydration, anti-wrinkle, and elasticity effects, there is still insufficient scientific evidence to confirm the efficacy of these HA-containing cosmetic products. Generally, high molecular weight HA (HMW-HA) plays an important role in skin hydration, osmotic balance, and the integrity of the ECM, while low molecular weight HA (LMW-HA) is associated with angiogenetic and pro-inflammatory activities (28, 29). However, HMW-HA has limited ability to penetrate the epidermis, particularly the lipid-rich stratum granulosum, which greatly restricts its topical benefits in hydrating the deeper layers of the epidermis (30). HA-based fillers represent another promising technique for skin rejuvenation. These fillers can be applied through mesotherapy, where HA is injected in small aliquots into various skin layers. However, a major limitation of injectable HA fillers is their rapid degradation by hyaluronidase, which limits their long-term efficacy (31). In addition, the injection of exogenously synthesised HA molecules can lead to a range of adverse effects, including immune response, oedema, ecchymosis, hyperaemia, and lumpiness (32). Therefore, strategies that focus on maintaining the balance of endogenous HA metabolism during photoaging may offer a new promising approach to overcome these limitations.

Gene therapy using siRNAs has emerged as a promising novel approach to treat a wide range of diseases. To maintain the balance of HA in the skin, we used siRNAs targeting HYAL2, which is significantly increased during photoaging. However, a major challenge in siRNA therapy is ensuring the efficient delivery of the siRNAs to target tissues. EVs have gained significant attention as bona fide carriers for siRNAs. EVs loaded with therapeutic siRNAs can easily be obtained on an industrial scale (33). When siRNAs are synthesized and transfected into donor cells, they can be efficiently released via EVs, which can be isolated from the culture medium through ultracentrifugation (33). EVs not only deliver intact siRNAs to target tissues but also, as natural biological materials, tend to induce fewer immune responses compared to synthetic carriers. For rejuvenating aged skin, topical delivery of siRNAs is the most practical approach. In this study, we selected ADSCs as donor cells to deliver siRNAs to the skin in a paracrine manner. ADSCs are well known for their regenerative potential, primarily through the paracrine secretion of various cytokines. Furthermore, ADSC-derived EVs have been reported to be effective paracrine executors of ADSC functions (34). By using ADSC-derived EVs as carriers, we not only specifically regulate HA metabolism through siRNA delivery but also introduce the additional benefits of ADSC-EVs on skin health. The combined effects of siRNAs and ADSC-EVs can significantly enhance skin rejuvenation, as demonstrated in our study. Moreover, ADSCs and their EVs are known for their high immune compatibility, which may reduce the risk of complications compared to the injection of synthesised chemical products (34). When administered subcutaneously, ADSCs efficiently regulate HA degradation in the dermis and the epidermis, indicating that ADSC-derived EVs can effectively traverse the different layers of skin and deliver their therapeutic cargo.

Regulating endogenous HA metabolism extends beyond cosmetic applications. This strategy is also applicable to other biological processes that rely on HA content, such as hydration, joint lubrication, and space-filling capacity. Our study provides a proof-of-concept strategy for modulating endogenous HA levels, with potential benefits that extend beyond skin rejuvenation to a wide range of physiological functions.

## Data Availability

The raw data supporting the conclusions of this article will be made available by the authors, without undue reservation.
