# Supplementary material for: Protecting and rejuvenating ageing skin by regulating endogenous hyaluronan metabolism using adipose-derived stem cell-secreted siRNAs
Source: Front Med (Lausanne). 2025 Apr 29;12:1529936. doi: 10.3389/fmed.2025.1529936 (PMC12069053; doi:10.3389/fmed.2025.1529936)
Supplement: Supplementary file 1 [file Table_1.docx]

**Protecting and rejuvenating skin aging by regulating endogenous hyaluronan metabolism using adipose-derived stem cell-secreted siRNAs.**

**Supplementary materials:**

**Figure S1:**

**
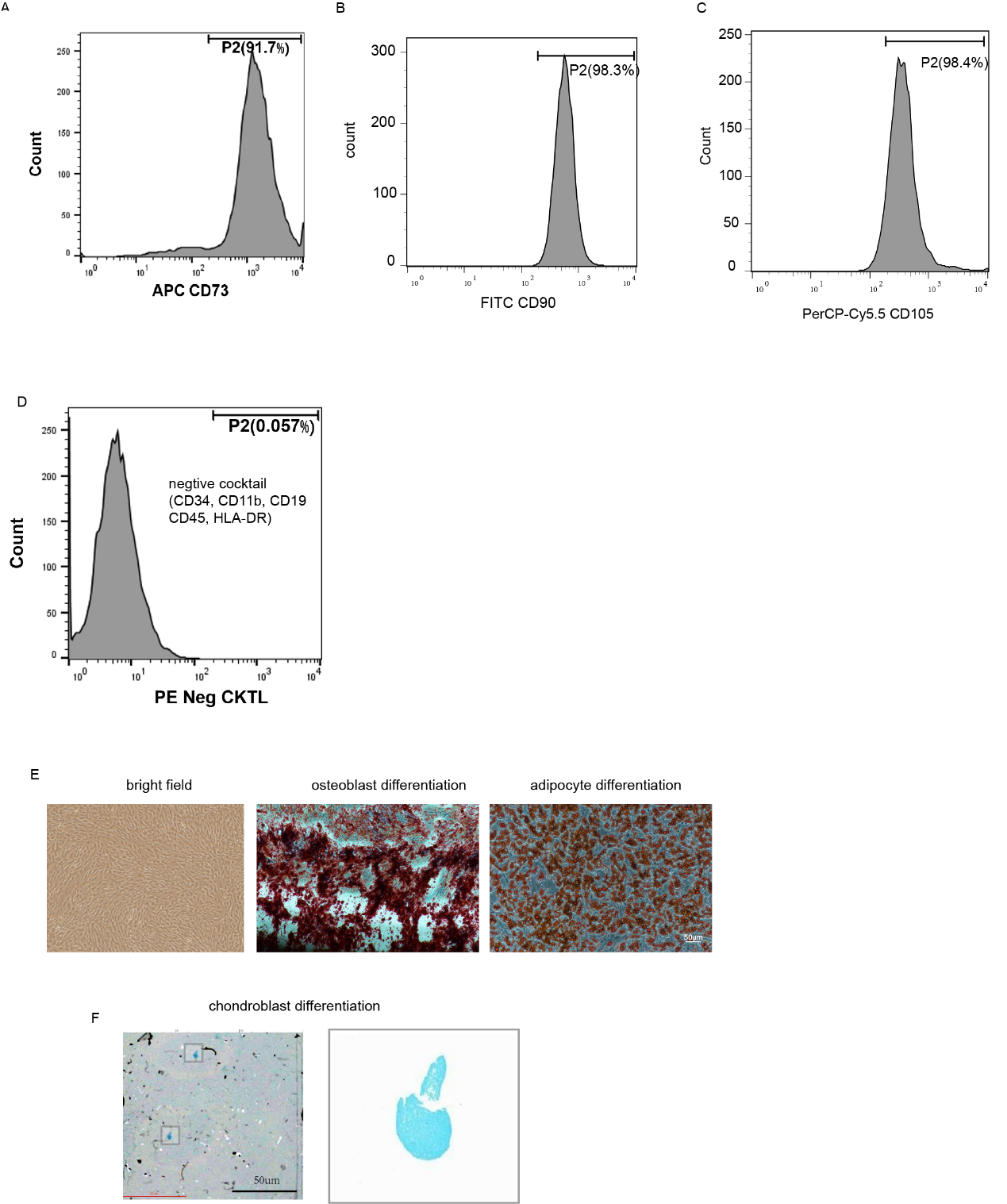
**

**Figure S1.** (A,B,C) Respective flow cytometry plots showing expressions of ADSC markers， including CD73 (A), CD90(B), and CD105(C). (D)The flow cytometry plots showing a panel of negative markers of ADSCs, including CD34, CD11b, CD19, CD45 and HLA-DR. (E) The bright field image of ADSCs, the osteoblast differentiation image of ADSCs, and the adipocyte differentiation image of ADSCs. (F) The chondroblast differentiation image of ADSCs.

**Figure S2:**

**
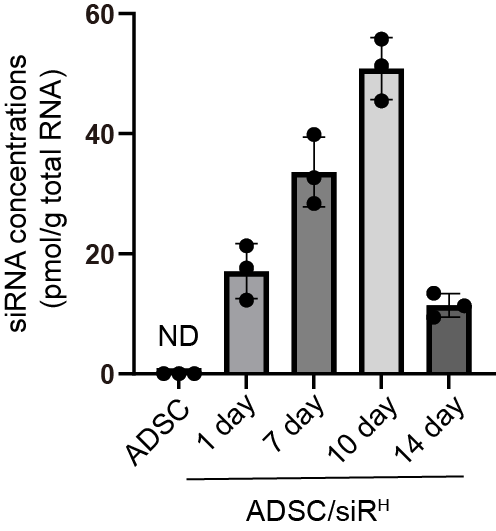
**

**Figure S2.** The siRNA concentrations in mice skins with the treatment of ADSC/siR^H^ or ADSCs in two weeks (n = 3).

**Primers and sequences:**

**Primers:**

HYAL2-fwd: 5’- CTTTGTCAGTTTCCGTGTTCG-3’

HYAL2-rev: 5’-CTTTGTCAGTTTCCGTGTTCG-3’

18s-fwd: 5’- CGGCTACCACATCCAAGGAA-3’

18s-rev: 5’- GCTGGAATTACCGCGGCT-3’

siRNAs sequences:

siRNA-hyal2-1: 5’- GGCCAUCAGACAGAGUAAUTT-3’

siRNA-hyal2-2: 5’- CUGCUACAAUCACGAUUAUTT-3’

siRNA-hyal2-3: 5’- GCCAAUACCUCAAGAAUUATT-5’
